# Facial EMG sensing for monitoring affect using a wearable device

**DOI:** 10.1038/s41598-022-21456-1

**Published:** 2022-10-07

**Authors:** Martin Gjoreski, Ivana Kiprijanovska, Simon Stankoski, Ifigeneia Mavridou, M. John Broulidakis, Hristijan Gjoreski, Charles Nduka

**Affiliations:** 1 Faculty of Informatics, Università della Svizzera italiana, 6900 Lugano, Switzerland; 2 Emteq Ltd., Brighton, BN1 9SB UK; 3 Faculty of Electrical Engineering and Information Technologies, Ss. Cyril and Methodius University in Skopje, 1000 Skopje, North Macedonia

**Keywords:** Computer science, Information technology

## Abstract

Using a novel wearable surface electromyography (sEMG), we investigated induced affective states by measuring the activation of facial muscles traditionally associated with *positive* (left/right orbicularis and left/right zygomaticus) and *negative* expressions (the corrugator muscle). In a sample of 38 participants that watched 25 affective videos in a virtual reality environment, we found that each of the three variables examined—subjective valence, subjective arousal, and objective valence measured via the validated video types (positive, neutral, and negative)—sEMG amplitude varied significantly depending on video content. sEMG aptitude from “positive muscles” increased when participants were exposed to *positively valenced stimuli* compared with *stimuli that was negatively valenced*. In contrast, activation of “negative muscles” was elevated following exposure to *negatively valenced stimuli* compared with positively valenced stimuli. High arousal videos increased muscle activations compared to low arousal videos in all the measured muscles except the corrugator muscle. In line with previous research, the relationship between *sEMG amplitude* as a function of subjective valence was V-shaped.

## Introduction

Remote solutions for timely detection and improved management of mood disorders could positively impact the lives of over one hundred million people in the EU alone and reduce healthcare costs. In 2018, the European Commission reported that a hundred million Europeans were experiencing mental health problems, including anxiety disorders, depression, drug/alcohol use, and bipolar disorder. Associated costs amounted to €600 billion, more than 4% of the EU’s GDP^1^. This situation was further aggravated by the COVID-19 pandemic, which has contributed to an increased prevalence of mental ill-health^2^. The need for robust affect analysis systems for researchers and healthcare professionals is clear.

Modeling emotions using a circumflex model of core affect^3^ involves recording markers or “fingerprints” of arousal and valence. Such markers include measurable psychological and physiological changes in the body during an emotional response^4^. For example, the fear response tends to combine activation of the motor functions, the autonomic nervous system (often linked to psychological arousal), and specific facial muscle activation (often linked to emotional valence). These markers are then mapped dimensionally in the arousal-valence 2D space. Affect monitoring systems have been developed using data from physiological sensors, including electroencephalogram (EEG) sensors, galvanic skin response (GSR) sensors, electrocardiogram (ECG) sensors, electromyography (EMG) sensors, electroencephalogram (EEG) sensors, voice analysis, and video analysis^5–11^.

Our face is considered one of the primary emotion expression mediators, and as such, it has been explored as the primary marker of valence^12^. Relevant to the dimensional model of affect, the zygomaticus major and the corrugator muscles have been extensively investigated to differentiate positive and negative valence, due to the muscle's role in smiling and frowning respectively^13,14^. There is, however, an ongoing debate as to whether reading from those muscles alone can suffice for valence detection or if other muscle groups should also be added^15,16^. The frontalis or “brow” muscle is responsible for the rising movement of our eyebrows, stretching on top of our forehead. Such movements are attributed to dynamic expressions of generalized fear, anger, and surprise, which could be of ambiguous valence. The measurement of the activation of multiple facial muscles in parallel could allow the discrimination between facial activations from which affective states could be inferred.

Surface EMG (sEMG) has frequently been used to measure muscle contractions using sensors applied directly on the skin. sEMG detects changes in surface voltages on the skin when muscle activation occurs. In part due to its ability to be applied non-invasively and high temporal sensitivity, facial sEMG has been used extensively to measure facial expression-derived emotional state^17,18^. However, such systems are not without their limitations. Traditionally, tethered, adhesive-based sensors often require the application of conductive gel, or the use of electrodes ill-suited to recording from what are relatively small muscles in the face^19^. This can lead to long setup types and electrodes becoming detached from skin.

**Figure 1:**
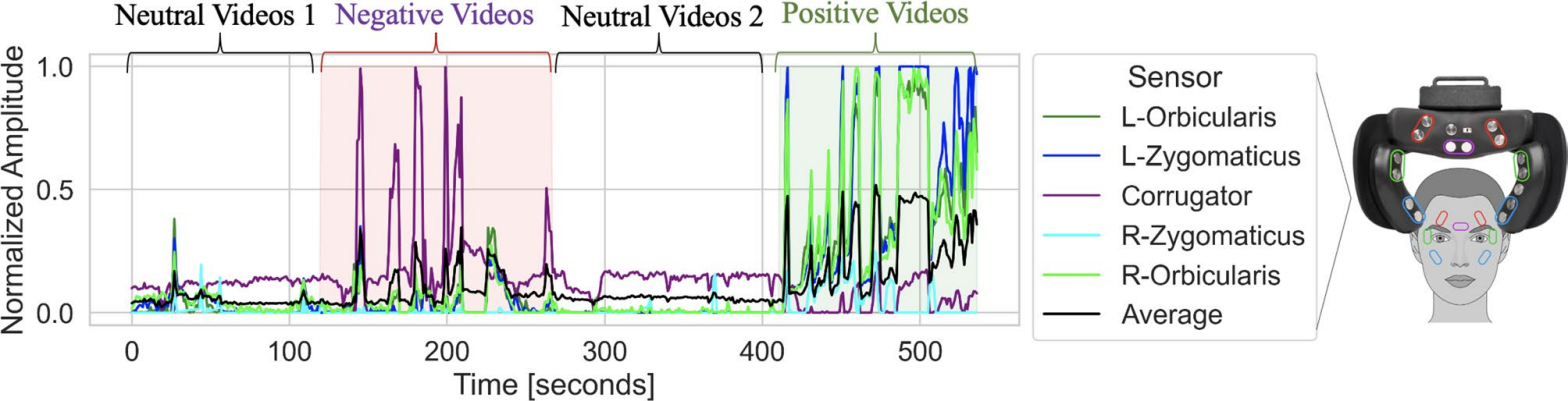
Example experimental session and sensor data collected for one subject. The lines represent the normalized sensor amplitudes for the five EMG sensors (left orbicularis, left zygomaticus, corrugator, right zygomaticus, and right orbicularis) and their average value. The session consists of 25 short videos.

This study investigates the association between facial sEMG and subjective and objective affect, using a novel wearable sEMG sensing device in VR environments. We used a set of dry, arrayed sEMG sensors (emteqPro)^20–22^, attached to a Pico Virtual Reality (VR) headset. During the experiments, physiological recordings and subjective self-reported data were collected from participants following exposure to videos varying in arousal and valence. Left and right-lateralized EMG activation readings from sensors overlapping the orbicularis, zygomaticus, and corrugator muscles were recorded (Fig. 1). The study had two objectives. The *first* was to explore the relationship between facial EMG activation, the emotional content of the video (arousal/valence) and self-reported arousal and valence provided retrospectively after each video clip. The *second* was to explore the relationship between facial EMG activations and a predefined categorical type rating for each video (either “positive”, “negative” or “neutral”).

The experimental results showed that for each of the three conditions examined (subjective valence, subjective arousal, predefined video type), sEMG signal amplitude varied depending on the video affective content. Facial muscle activation associated with “positive expressions” (i.e., smiling) increased in periods with *positively valenced content* compared with *negatively valenced content*. In contrast, facial muscle activation associated with “negative expressions” (i.e.,* frowning*) was higher during periods with *negatively valenced stimuli* compared to periods with *positively valenced stimuli*. High arousal videos induced increased muscle activations compared to low arousal videos in all the measured muscles except the corrugator muscle. For the corrugator muscle, some level of activation was observed both during the low arousal and during the high arousal videos.

These results confirmed the link between facial muscle activation and subjective and objective affect, while also demonstrating that dry EMG sensor fixed in a pre-set array can be used to monitor affective states in VR environments. These conclusions contribute to affect sensing in VR, which has many potential use-cases, including symptom monitoring during VR-delivered therapy, improved affect analytics in VR gaming, affective Human–Computer Interaction (HCI), and improved avatar-based social interactions in the metaverse^23^.

## Results

### Experimental validity

In the study, 25 videos were presented to 38 participants. The videos were selected to induce neutral valence with low-arousal (‘neutral’), positive valence with high-arousal (‘positive’), and negative valence with high-arousal (‘negative’). After each video, participants were asked to self-report subjective arousal and valence (on a scale from one to nine). Those were recorded and combined to test the effects of the affective video stimuli on the participants’ affect (manipulation check).

**Figure 2:**
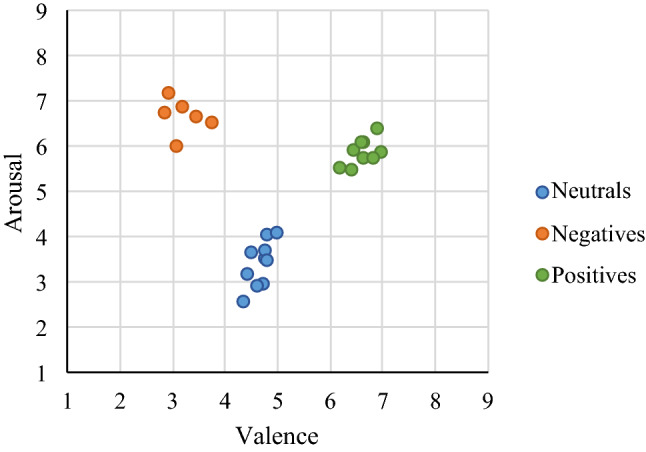
Average valence and arousal scores per video visualized on the 2-Dimensional affective space.

Table 1 shows the mean scores per video category. Figure 2 shows the average score for each video presented. As illustrated, a consistent differentiation can be seen between each of the three video categories.

**Table 1:** Average self-reported valence and arousal scores per video category.

	Valence	Arousal
Mean neutral	4.65 ± 1.11	3.41 ± 1.65
Mean negative	3.18 ± 1.70	6.67 ± 1.40
Mean positive	6.60 ± 1.26	5.88 ± 1.48

Following tests for normality, the self-ratings were then analyzed using Friedman’s tests. Results are described firstly for valence scores and then for arousal. Significant results were followed with Wilcoxon’s tests.

#### Valence scores

Friedman’s tests showed a significant difference between mean subjective valence scores of the three conditions, (neutral, negative, and positive; ch2 (2) = 67.46, *p* < 0.001). The mean rank for the neutral category was 1.81, the mean rank for the negative category: was 1.21, and the mean rank for the positive category: was 2.98. Wilcoxon tests showed that valence scores in the negative category were lower than in the neutral category (z = 4.664, *p* < 0.001), and positive category (z = 5.602, *p* < 0.001). The positive category was rated the highest and showed a significant difference to the neutral category (z = 5.646, *p* < 0.001).

#### Arousal scores

Friedman’s tests showed a significant difference between the mean subjective arousal scores for the three conditions: neutral, negative, and positive (ch2 (2) = 62.65, *p* < 0.001). The mean rank for the neutral category was 1.05, the mean rank for the negative category: 2.71, and the mean rank for the positive: 2.24. As with the valence scores, all categories were found to differ significantly (Negative–Neutral: z = − 5.644, *p* < 0.001, Positive–Negative: z = − 3.71, *p* < 0.001, Positive-Neutral: z = − 5.62, *p* < 0.001).

Results appeared to confirm that all video categories were significantly different from each other in terms of self-reported arousal and valence. Each video category succeeded in inducing the intended valence and arousal in the participant (neutral valence with low-arousal for the ‘neutral’ category, positive valence with high-arousal for the ‘positive’ category, and negative valence with high-arousal for the ‘negative’ category). We concluded that the affect manipulation check was successful, and the self-ratings, together with the video category names (video types), were used as ground truth for further analysis.

Is the wearable EMG sensing device appropriate for differentiating positive versus negative subjective valence?

**Figure 3:**
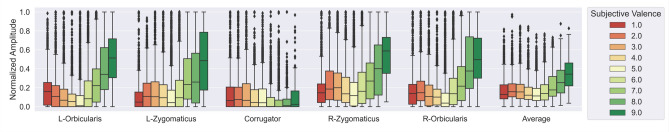
Boxplots representing the distribution of the normalized EMG amplitudes for the five EMG sensors (left orbicularis, left zygomaticus, corrugator, right zygomaticus, and right orbicularis) and their average amplitude, grouped by subjective valence ratings from one (negative valence) to nine (positive valence).

Figure 3 shows normalized sEMG amplitudes. The x-axis contains six subgroups, the five EMG sensors (from left to right: left orbicularis, left zygomaticus, corrugator, right zygomaticus, and right orbicularis) and the average of the five EMG sensors. Within each EMG sensor, the data is also grouped per subjective valence levels, from one (negative valence) to nine (positive valence). The valence levels are also color-coded.

For the orbicularis muscle (L-Orbicularis in Fig. 3), the mean signal amplitude is around 0.20 for the videos ranked with the lowest valence level (left most, red boxplot). Signal amplitude decreases following exposure to video content viewed subjectively as “neutral” (i.e., self-report of “five” on a nine-point scale of subjective valence). As subjective valence increases from neutral to positive (i.e., shifts from six to nine), there is a corresponding increase in left-orbicularis sensor activation. The mean amplitude for the two final valence levels (eight and nine) is between 0.30 and 0.50, higher than the mean amplitude for the negative valence (between 0.10 and 0.20). This suggests amplitude of the left orbicularis sensor is lowest for the videos rated as neutrally valanced. Compared to the videos with neutral valence ratings (e.g., five), left orbicularis muscle activation increases to 0.10 and 0.20 for the videos ranked with negative and reaches its maximum for those ranked with positive valence levels. Such V-shaped activation can be observed for all other sensors (left/right orbicularis and left/right zygomaticus), notably except for the sensor overlapping the corrugator muscle. For the corrugator, EMG amplitude is highest for videos judged to be negatively valenced, and lowest for videos judged positively valenced. Furthermore, for the videos ranked with valence level 5 (i.e., neutrally valenced), the amplitude of the corrugator sensor stays quite high, achieving similar activation as some of the negative valenced videos.

**Figure 4:**
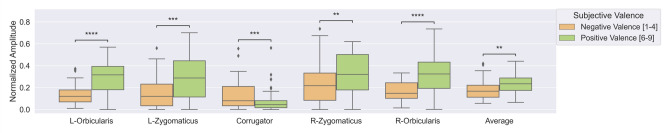
Wilcoxon signed-rank (paired) test with Bonferroni correction for videos with negative valence ratings (1–4) versus videos with positive valence ratings (6–9), applied for each of the five sensors (left orbicularis, left zygomaticus, corrugator, right zygomaticus, and right orbicularis) and the average amplitude of all five sensors. Statistical significance annotations: * if *p* ∈ [.05, 10^−2^); ** if *p* ∈ [10^−2^, 10^−3^); *** if *p* ∈ [10^−3^, 10^−4^); and **** if *p *≥ 10^−4^.

To perform statistical tests, we split videos according to subjective ratings of valence. Those that received subjective ratings of 1–4 were categorized as “negative videos” and those that received subjective ratings of 6–9 were categorized to be “positive videos”. For each of the five sensors, we calculated the mean normalized EMG signal amplitude $\overline{{M\left( {s, V_{l} } \right)}}$, for each video ranked with valence level $V_{l }$ (where $V_{l}$ can be $V_{negative}$ or $V_{positive}$), for each subject s. Thus, if the subject ranked several videos with valence level $V_{l}$, the mean amplitude was calculated over all those videos. This procedure led to n = 38 paired samples (one pair [positive, negative] amplitude per subject), where one component of the pair represents the mean sensor amplitude for negative valenced videos, and the other component of the same pair represents the mean sensor amplitude for positive valenced videos. To test whether there is a statistically significant difference in the mean amplitudes of each video type, we used the Wilcoxon signed-rank (paired) test with an alpha level of 0.05, FamilyWise Error (FWE) corrected using a Bonferroni correction for the six tests (one test for each of the five sensors and another one for their average). Results are presented in Fig. 4 which shows all the five sensors and their averaged values. There are statistically significant differences among the normalized EMG amplitudes. For negative videos (i.e., valence ratings of 1–4), the amplitudes of the sensors left/right orbicularis and left/right zygomaticus are lower, whereas the amplitude of the corrugator sensor is higher. On the other hand, for the positive valenced videos, the relation is reversed.

Is the wearable EMG sensing device appropriate for differentiating low versus high subjective arousal?

**Figure 5:**
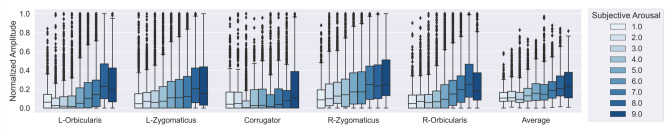
Boxplots representing the distribution of the normalized EMG amplitudes for the five EMG sensors (left orbicularis, left zygomaticus, corrugator, right zygomaticus, and right orbicularis) and their average amplitude, grouped by subjective arousal ratings from one (low arousal) to nine (high arousal).

Figure 5 presents the distributions of the normalized EMG amplitudes via boxplots. The x-axis contains six subgroups, the five EMG sensors (from left to right: left orbicularis, left zygomaticus, corrugator, right zygomaticus, and right orbicularis) and the average of the five EMG sensors. Within each EMG sensor, the data is also grouped per subjective arousal level, from one (low arousal) to nine (high arousal). The arousal levels are also color-coded. As reported in the boxplot of the left orbicularis muscle (L-Orbicularis in the figure), the mean amplitude is below 0.10 for the videos ranked with the lower levels of arousal (e.g., from one to four). As the arousal level increases from low to high (e.g., above six), the amplitude of the sensor also increases, reaching mean values just above 0.20. This relation, i.e., low activation for low arousal versus increased activation for high arousal, can be observed for all the sensors except the corrugator sensor. For the corrugator, the amplitude has similar levels across all videos, regardless of the arousal levels.

**Figure 6:**
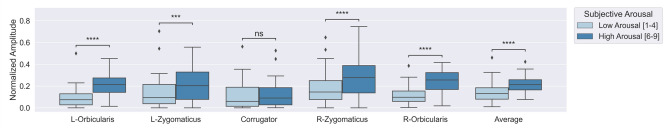
Wilcoxon signed-rank (paired) test with Bonferroni correction for videos with low arousal ratings [1–4] versus videos with high arousal ratings [6–9], applied for each sensor (left orbicularis, left zygomaticus, corrugator, right zygomaticus, and right orbicularis) and their average amplitude. Statistical significance annotations: * if *p* ∈ [.05, 10^−2^); ** if *p* ∈ [10^−2^, 10^−3^); *** if *p* ∈ [10^−3^, 10^−4^); and **** if *p* ≥ 10^−4^.

Similar to the subjective valence analysis, to perform statistical tests, we split videos judged to be low (subjective ratings 1–4) and high (subjective ratings 6–9) into two groups. For each of the five sensors and their averaged values, we calculated the mean normalized amplitude $\overline{{M\left( {s, A_{l} } \right)}}$, for each video ranked with arousal level $A_{l}$ (where $A_{l}$ can be $A_{low}$ or $A_{high}$) for each subject s. Thus, if the subject ranked several videos with arousal level $A_{low}$, the mean amplitude was calculated over all those videos. This procedure led to n = 38 paired samples (one pair per subject), where one component of the pair represents the mean sensor amplitude for low arousal videos, and the other component of the same pair represents the mean sensor amplitude for high arousal videos. To test whether there is a statistically significant difference in the mean amplitudes for low versus high arousal videos, we used the Wilcoxon signed-rank (paired) test with an alpha level of 0.05, FWE corrected using a Bonferroni correction for the six tests (one test for each of the five sensors and another one for their average). The results of these experiments are presented in Fig. 6. As illustrated, there are statistically significant differences in five of the six tests performed. For the low arousal videos, the amplitudes are lower, whereas for the high arousal videos, the amplitudes are higher. Only for the corrugator sensor, there is no statistically significant difference between low and high arousal videos.

Is the wearable EMG sensing device appropriate for distinguishing positive versus neutral versus negative videos?

In the previous analyses (Figs. 5 and 6), we explored the relationship between the EMG amplitudes and the subjective video ratings (arousal and valence). In the following analysis, we will explore the relationship between the EMG amplitudes and the type of the videos (positive, neutral, or negative).

**Figure 7:**
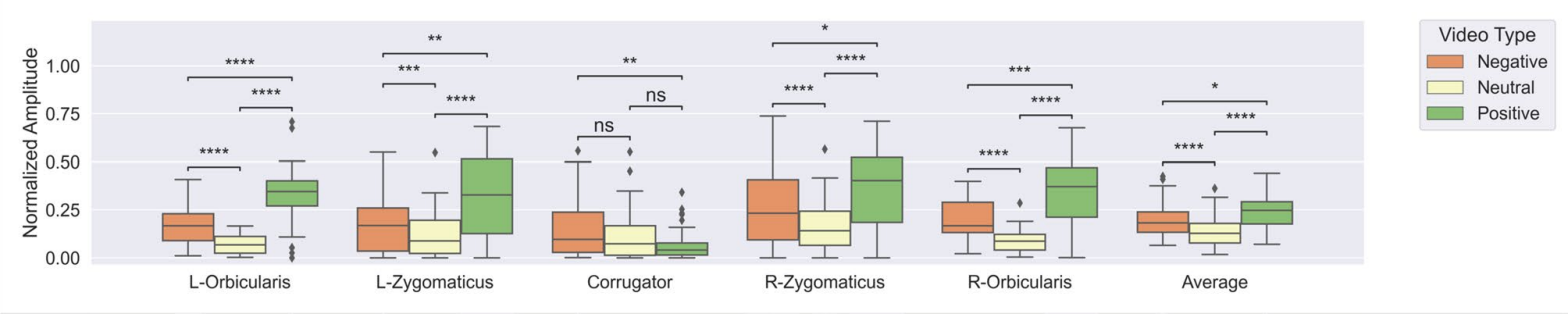
Wilcoxon signed-rank (paired) test with FWE corrected for each sensor (left orbicularis, left zygomaticus, corrugator, right zygomaticus, and right orbicularis) and their average amplitude for comparing: neutral versus positive, neutral versus negative and positive versus negative videos. Statistical significance annotations: * if *p* ∈ [.05, 10^−2^); ** if *p* ∈ [10^−2^, 10^−3^); *** if *p* ∈ [10^−3^, 10^−4^); and **** if *p* ≥ 10^−4^.

For each of the five sensors and their averaged values, we calculated the mean normalized amplitude $\overline{{M\left( {s, W_{l} } \right)}}$, for each video type $W_{l}$ (where $W_{l}$ can be $W_{negative} , W_{neutral} ,$ and $W_{positive}$), for each subject s. The calculated values represent the mean amplitude levels per subject, measured while the subjects were watching a video of a specific type. This procedure led to n = 38 tuples of three values. One value represents the mean sensor amplitude for neutral videos, another represents the mean sensor amplitude for positive videos, and the third represents the mean sensor amplitude for negative videos. To test whether there is a statistically significant difference in the mean amplitudes, for all pairs of videos—neutral versus positive, neutral versus negative, and positive versus negative—we used the Wilcoxon signed-rank (paired) test with Bonferroni correction for 18 tests (3 pairs of videos × 6 sensing modalities, the five sensors and their average). The results of these experiments are presented in Fig. 7. From the figure, it can be seen that the difference in the EMG amplitudes is statistically significant in the majority of the cases (16 out of the 18 paired tests). The two tests for which the difference was not statistically significant were the tests comparing neutral versus positive and neutral versus negative videos using the data from the corrugator muscle. Inversely to those two tests, for the test comparing positive versus negative videos using data from the same sensor (corrugator), the difference was statistically significant—the negative videos caused higher corrugator activation than the positive videos. From the tests applied over the data of the left/right orbicularis and left/right zygomaticus sensors, it can be seen that the amplitude of the sensors is lowest for the neutral videos, with mean values below 0.20. The activation increases to 0.20 for the negative videos, reaching the highest levels for the positive videos (e.g., above 0.3). This V-shaped activation was also observed in the analysis of the subjective valence ratings (Fig. 3).

## Discussion

### On the V-shaped relation between valence and arousal

It is not a surprise that positively valenced videos (e.g., babies, cats, and dogs), induce positive emotion compared with negatively valenced videos. This finding was confirmed for the 25 affective videos used in our study. On a valence scale between one and nine, negative videos had a mean score of 3.18, neutral videos of 4.65 and positive videos of 6.60. See Table 1 and Fig. 2 for more details. Furthermore, on an arousal scale between one and nine, the mean scores were 6.67 (negative videos), 3.42 (neutral videos), and 5.88 (positive videos). This V-shaped relation between valence and arousal, i.e., the valence level increases, and the corresponding arousal starts at a *higher* level for the negative videos, then *decreases* for the neutral videos, and finally *increases* again for the positive videos, is in line with related affect studies performed on larger cohorts. For example, Kuppens et al.^27^ examined eight affective datasets (with the number of participants ranging from 80 to 1417) and concluded that, on average, there is a weak but consistent V-shaped relation of subjective arousal scores as a function of subjective valence scores.

Interestingly, besides the V-shaped relation between the self-reported ratings, our experiments also depicted similar V-shaped relation of the EMG facial amplitudes as a function of valence. This relation can be seen both for self-reported (Fig. 3) and “objective” valence (type of the videos, Fig. 7). If we consider that subjective arousal is related to the intensity of the affective state and that facial EMG amplitude is related to the intensity of the muscle activation, then again, this relation comes as no surprise. Similar J-shaped activation for the zygomaticus has been reported in related studies that use traditional EMG sensing^14,28^.

**Figure 8:**
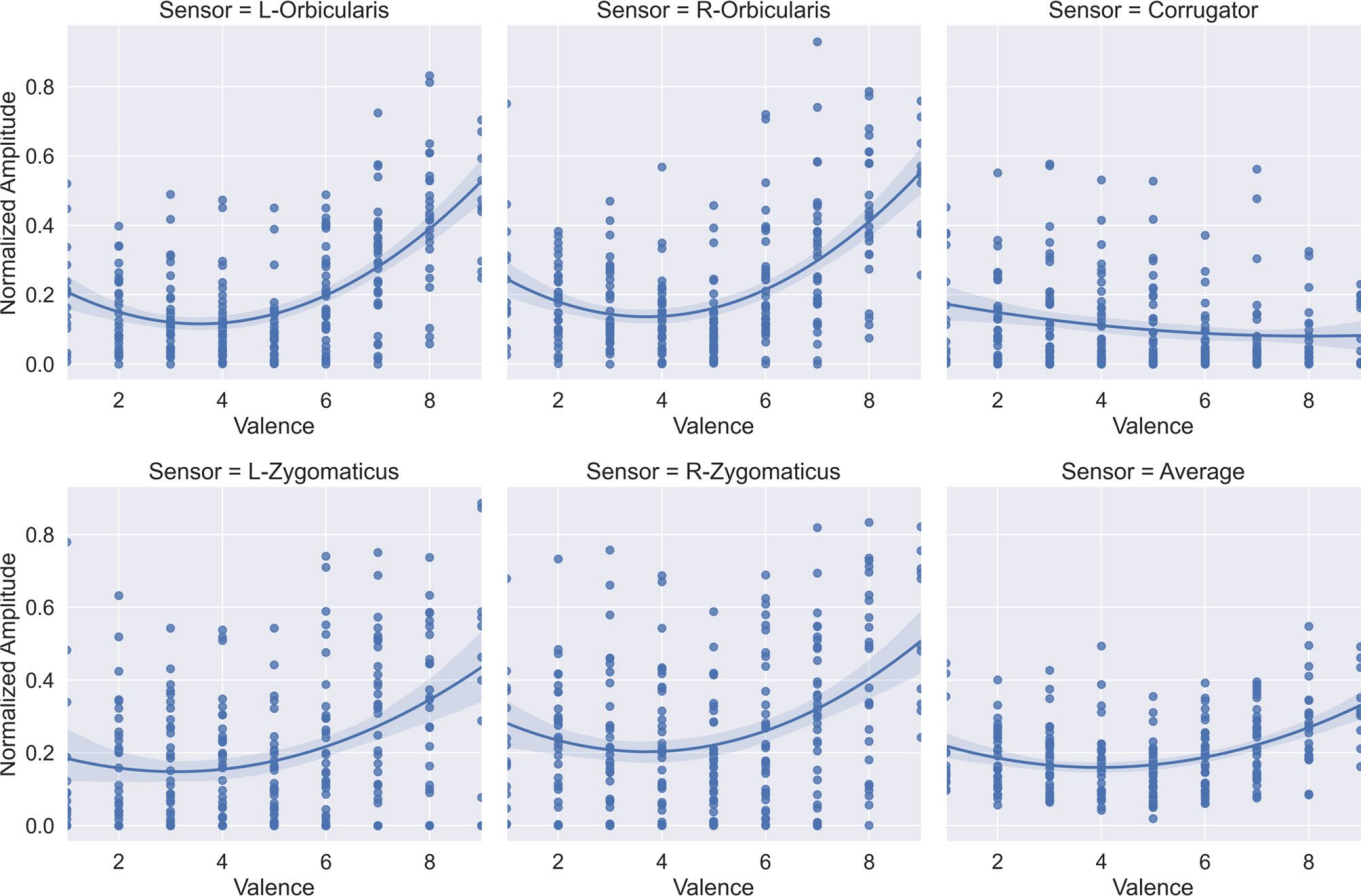
V-shaped relation between the subjective valence ratings (y-axis) and sEMG amplitudes (x-axis). Each scatterplot corresponds to one sensor (left/right orbicularis, corrugator, left/right zygomaticus, and the average of the five sensors).

To explore this V-shaped relation, we present six scatterplots in Fig. 8 (one scatterplot per sensor), and we present corresponding Pearson’s correlation coefficients (PCCs) in Table 2. In each scatterplot, the x-axis shows subjective valence ratings, the y-axis, normalized amplitude. Each plot also contains a second-order regression model fitted over the data points in each scatterplot. The translucent bands around the regression lines show 95% confidence interval. One data point represents one user’s average amplitude value at the given valence level. The V-shaped relation is visible through the regression lines for the left/right orbicularis, the left/right zygomaticus, and the average of the five sensors. The regression line for the corrugator muscle monotonously decreases as the subjective valence increases.

**Table 2:** Pearson’s correlation coefficient (and corresponding statistical significance in brackets) for the relation between subjective valence and sEMG amplitudes.

Subjective valence	Orbicularis		Zygomaticus		Corrugator	Sensors average
	Left	Right	Left	Right		
Negative–Neutral [1–5]	− 0.24 (**)	− 0.27 (**)	− 0.06 (n.s.)	− 0.11 (n.s.)	− 0.13 (n.s.)	− 0.2 (*)
Neutral–Positive [5–9]	0.64 (***)	0.61 (***)	0.41 (***)	0.45 (***)	− 0.02 (n.s.)	0.53 (***)
Negative–Positive [1–9]	Not suitable for correlation analysis (non-linear relationship)				− 0.20 (***)	–

The subjective valence is represented in three variations: Negative to Neutral [1–5], Neutral to Positive [5–9], and Negative to Positive [1–9]. Statistical significance annotations: * if *p* ∈ [.05, 10^−2^); ** if *p* ∈ [10^−2^, 10^−3^); *** if *p* ∈ [10^−3^, 10^−4^); and **** if *p* ≥ 10^−4^.

Significant values are in [bold].

To quantity the slopes of the V-shaped relation, we calculated PCCs (and corresponding *p*-values in brackets) in three conditions (see Table 2):

- *Negative to Neutral [1–5]—*to quantify the left part of the V-shape, i.e., negative to neutral valence, we calculated the PCC between the sEMG amplitudes and the subjective valence scores from one to five (including one and five). For the orbicularis muscles, the results suggest a weak (*r-left* = − 0.24 and *r-right* = − 0.26), but statistically significant (*p* ≤ 0.001) negative relationship. For the rest of the muscles, the relationship is not statistically significant.
- *Neutral to Positive [5–9]—*to quantify the right part of the V-shape, i.e., neutral to positive valence, we calculated the PCC between the sEMG amplitudes and the subjective valence scores from five to nine (including 5 and 9). In these tests, there is a strong (*r* > 0.40) and statistically significant (*p* ≤ 0.001) positive correlation.
- Negative to Positive *[1–9]*—to quantify the linear relationship for the corrugator muscle, we calculated the PCC between the sEMG amplitudes and the subjective valence scores from one to nine. The results suggest a weak (*r* = − 0.20), but statistically significant (*p* ≤ 0.05) relation.

Additionally, the Supplementary Fig. S.1 presents the results of the statistical tests performed with 3-class subjective valence (negative vs. neutral vs. positive). The results from Supplementary Fig. S.1, and the results from Fig. 8 and Table 2, point out to similar conclusions, i.e., the V-shaped relation between *subjective* valence and sEMG data is more pronounced for the orbicularis data. Nevertheless, to the best of our knowledge, this is the first study that reports on the V-shaped relation of EMG facial amplitude measured via sEMG as a function of subjective (Fig. 8) and objective (Fig. 7) valence both for the left/right orbicularis and for the left/right zygomaticus. The corrugator muscle showcases an inversed linear relation between the valence and EMG amplitude, which is also related to more traditional EMG-sensing studies^14^.

### On the study objectives

For subjective valence, activation of the muscles traditionally associated with positive expressions (left/right orbicularis and left/right zygomaticus) was increased in periods with high valence ratings compared to the activation of the same muscles during periods with lower valence ratings. In contrast, activation of the muscles associated with negative expressions, in our case the corrugator, was higher during periods with lower valence ratings compared with periods with higher valence ratings. These differences were statistically significant.

For subjective arousal, during periods that were rated with low arousal, the amplitudes of the sensors were overall lower, whereas during periods with high arousal, the amplitudes were higher. Except for the corrugator muscle, these differences were statistically significant for all tested modalities. For the corrugator sensor, there was no statistically significant difference between low and high arousal activations.

For the predefined video types, similar findings were observed, i.e., the activation of the muscles related to positive expressions (left/right orbicularis and left/right zygomaticus) were higher during the videos categorized as “positive” compared to the activation of the same muscles during videos categorized as “neutral” and “negative”. In contrast, activation of the corrugator muscle (typically associated with negative expressions like frowning), was higher during videos categorized as “negative” compared with “positive”. The differences were statistically significant.

All of the experimental scenarios for which statistical significance was not observed involved the corrugator muscle. One explanation for this is the fact that activation of the corrugator muscle is involved in both negative (e.g., frowning) and non-negative (e.g., concentrating/focused) facial expressions. An alternative or complimentary explanation could lay in the overlay of facial musculature. While the sEMG electrodes are positioned to maximize the quality of measurement of targeted muscles, facial musculature is complex, with several muscles overlaying one on top of another. Therefore, isolated measurements of the targeted muscles can’t be guaranteed simply because even relatively small electrodes can easily end up overlapping multiple muscles which may have opposing directions of travel. The corrugator muscle is positioned just below the frontalis muscle but is involved in very different facial expressions (i.e., frowning vs brow raised).

## Conclusions and implications for future work

We investigated the association between facial sEMG, subjective and objective affect, using a novel wearable sEMG sensing device in VR environments using a cohort of 38 participants that watched a selection of a total 25 affective video stimuli validated in independent studies^24,26^. The experimental results showed that for each of the three conditions examined (subjective valence, subjective arousal, predefined video type), sEMG signal amplitude significantly varied depending on the affective content of the video being watched. Activation of muscles associated with positive affect (left/right orbicularis and left/right zygomaticus) was increased during positive periods compared to negative periods. Similarly, activation of these muscles was higher during periods with high arousal compared to periods with low arousal.

Another finding was linked to the V-shaped relation between valence and arousal—a relation previously known in the context of *subjective* arousal and valence ratings^27^. J-shaped activation of the zygomaticus muscle has been reported also in traditional EMG studies^14,28^. Our experiments depicted similar relation of the sEMG facial amplitudes as a function of valence, not just for the zygomaticus muscles, but also for the orbicularis muscles.

The presented analysis depicts the average differences in sEMG amplitudes measured in a group of 38 participants when presented with affective video stimuli in a VR environment. The next step would be to move from the group-based analysis to a personalized and real-time analysis of facial expressions and affect. Such advanced analytics would include real-time data processing, dynamic segmentation, and feature extraction, coupled with machine-learning algorithms that can recognize affect at a high-frequency rate (e.g., 1 Hz). Another promising direction includes multimodal affect recognition. Although the face is known to be the richest source of valence information as well as affect as a whole^29^, there are additional physiological indicators that can provide affective information. Whenever users are exposed to an affective stimulus, their affect is also expressed through physiological cues including the activity of the heart, the brain, the body, and the eyes^30^. Also, facial expressions do not always reflect how users are feeling. Therefore, by using a multimodal approach, we can get a deeper understanding of how the users are responding to a given stimulus (e.g., video or mental health therapy). For example, when analyzing the nature of a particular video, e.g., positive or neutral, it is important to know if the user is focused on the part of the video that should elicit the expected affective response. For this purpose, by combining eye tracking with sEMG data, we could recognize more accurately the type of the video stimulus (e.g., positive, negative, or neutral). Group-based analysis should provide more objective recognition of video types, whereas personalized analysis should provide more information on user-specific preferences.

The findings of this study have a direct implication in current VR/Augmented Reality (AR) applications where scenario creators can benefit from automatically monitoring users’ affective responses (e.g., the entertainment industry). For example, VR/AR creators could automatically measure which segments of the content are exciting (e.g., higher arousal), which segments are boring (e.g., lower arousal), which segments induce positive affect (e.g., positive valence), and which segments induce negative affect (e.g., negative valence). Another more impactful use case involves remote solutions for mental health studies. Researchers and healthcare professionals could run longitudinal remote studies with mental-health patients. Such a study could monitor the users’ affective responses while performing some tasks (either in VR, or potentially VR/AR) specifically designed to help the users combat depression. The remote affective feedback from the sEMG sensors could be a real-time indicator of whether the tasks are helpful for fighting depression. Similar studies could be performed in VR/AR-based exposure therapies for combating phobias. Whichever the use case is, such affect monitoring systems should include ethics, fairness, model explainability, and accountability at their core.

## Methods

### Emotions and affect

Scientific research on emotions was introduced back in 1868 when Charles Darwin undertook a study to prove that humans have an innate and universal set of emotional expressions. In 1872 the study was published in his book “The Expression of the Emotions in Man and Animals”^31^. In 1987, the question “Can computers feel” was raised^32^. In the early 1990s, Picard published her book “Affective Computing”^33^, which many consider the start of this scientific field. Yet three decades after publication, with Affective Computing now a well-established research field, accurately modeling how a person feels remains a challenge.

When analyzing human emotions and affective states, two different approaches can be taken, a discrete approach or a continuous approach. In the discrete approach, the emotions are represented as discrete and distinct states, i.e., anger, fear, sadness, happiness, boredom, disgust, and neutral^34^. Although commonly used in commercial settings, a frequent failure in replication across individuals has called the universality of discrete emotions into question. A handful of recent meta-analyses on autonomic physiology, behaviors, and brain imaging, as well as electrical stimulation studies, intracranial recording studies, and brain lesion studies in humans, all report that, to date, no consistent and specific biological or behavioral fingerprints (i.e., no biological essences) for different categories of emotion, like anger, sadness, and fear^35,36^. Consequently, researchers have argued that emotions and emotional expressions are not universal^35,37^. Instead, most studies in Affective Computing utilizing multimodal methods analyze affective states using the dimensional approach^38^. Based on this approach, emotional states and their intensities are described as phenomenological reactions to an experience, modeled along a continuum. The most popular is the dimensional model of pleasantness (Valence), and activation (Arousal) model, or else Russel’s circumflex model of core Affect^3^. Arousal and valence are dissociable constructs that describe an experience by how positive (high valence) to negative (low valence) it is perceived, and intense that feeling was, ranging from soothing/calming (low arousal) to exciting/agitating (high arousal). Put together this dimensional approach has been widely used for the development and visualization of affect detection systems as well as for the annotation of user self-ratings which is still considered as ‘ground truth’^39–41^.

### Participants

A group of 38 healthy volunteers, 14 females and 24 males, with a mean age of 33.4 ± 13.6 were recruited to participate in the experiment. The inclusion age range was 16–68 years, but all recruited participants were above 18 years old. Detailed demographic information about the recruited participants is available in the Supplementary Table S.1. Exclusion criteria for recruitment were the presence of cardiovascular conditions and the use of medications. Ethical approval was obtained from the *Bournemouth University Ethics Committee on 30 November 2020 (Approval No. 33494)*. All participants also provided written informed consent before participating in the study. The experiment was conducted following institutional ethical provisions and the Declaration of Helsinki.

### Video Stimuli

We used 25 video stimuli to manipulate participants' affective states. A short description of the videos and the validation studies is provided in Supplementary Table S.2. A combination of video stimuli was selected from the public video library by Samson et al.^24^. The videos were validated by 411 subjects in that initial study, and additional validation with 82 participants was performed in another study^25^. For our study, the neutral, the high negative, and the high positive arousing videos were utilized. One negative video was added from a study by Gnacek et al.^26^, and seven new positive videos were introduced. The positive videos were introduced because the existing positive videos were of poor quality. Overall, the video selection was intended to elicit three distinct levels of valence responses (negative, neutral, and positive). Thus, a selection of videos was made per valence category. Six videos represented negative content, nine represented positive content, and ten represented neutral content. The ten neutral videos were randomly split into group one, shown at the beginning of the experiments, and group two, shown after the negative videos. The order of the videos was: five neutral (overall duration of 76 s), six negative (overall duration of 93 s), five neutral (overall duration of 81 s), and nine positive videos (overall duration of 78 s).

Additionally, we performed validation of the videos using the self-ratings reported by the participants recruited in this study. The total duration of the videos was 338 s, with a mean and standard deviation of 12.08 ± 8.17 s. All videos were displayed in a VR environment, resembling a VR home cinema. The VR environment contained a screen where the videos were presented, while low to medium brightness was chosen for the remaining room to avoid distraction.

### Apparatus

**Figure 9:**
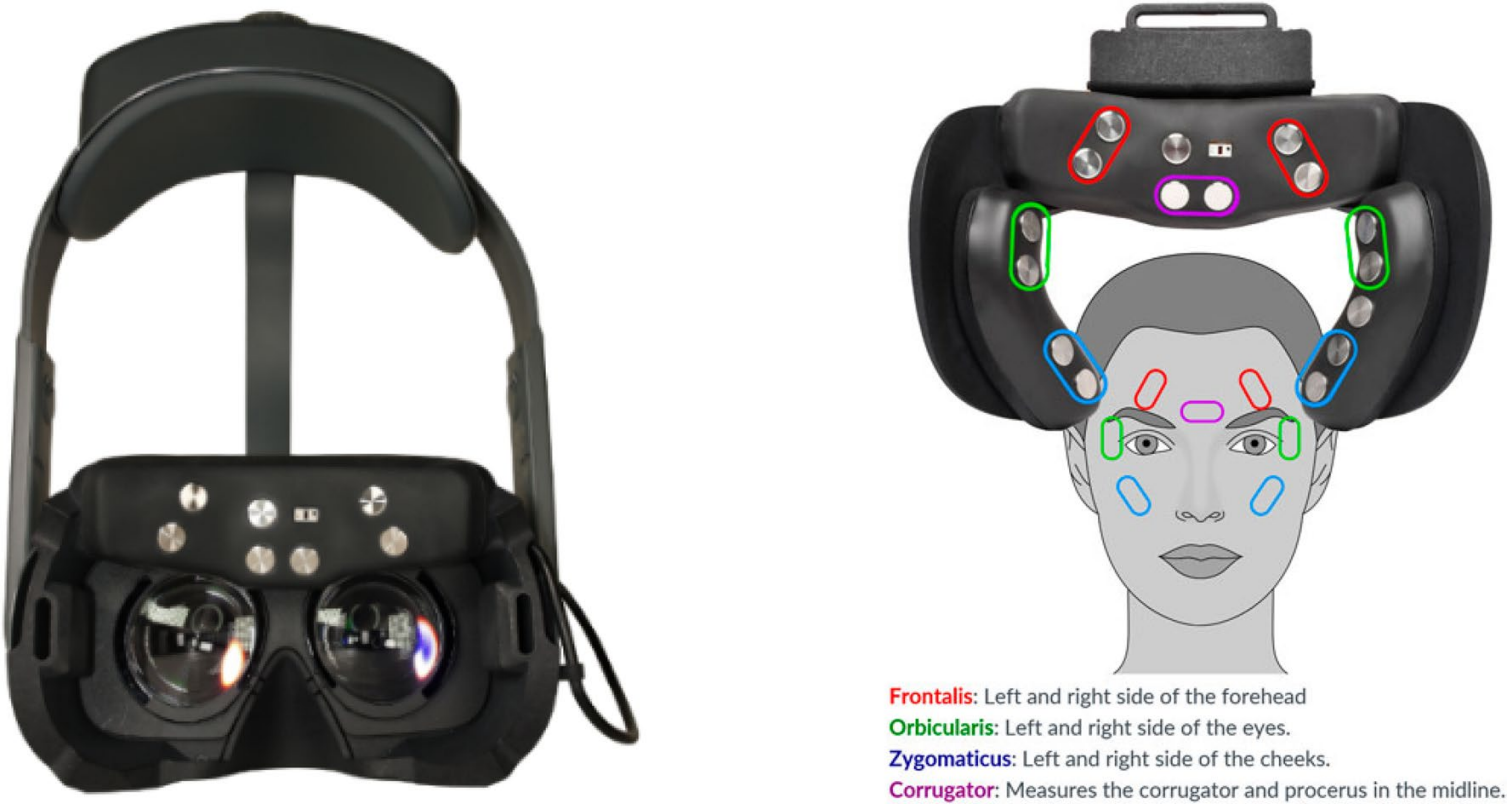
The figure on the left depicts the emteqPro mask installed into the Pico Neo 2 Eye Virtual Reality (VR) headset. The figure on the right depicts the mapping between emteqPro’s EMG sensors and facial muscles.

For data collection, we used the emteqPro device^20–22^. The device comprises a facial electromyographic (EMG), a photoplethysmographic (PPG), and an inertial measurement unit (IMU) sensor integrated within a soft frame that fits on the face of the wearer. The device was inserted into a Pico VR headset. The emteqPro mask incorporates seven facial EMG electrodes for capturing facial muscle activation. Those are positioned to overlap the frontalis (left and right side of the forehead), orbicularis (left and right side of the eyes), zygomaticus (left and right side of the cheeks), and corrugator muscles (Fig. 9). In this study, we analyzed only the data coming from the EMG sensors. Sensor data from the frontalis sensors was not included in this analysis.

### Experimental scenario

Prior to starting the data collection procedure, all participants were introduced to the experiment, the task, and the equipment. Then, they were asked to remove any skin products (i.e., make-up) or hair from the face to ensure good sensor contact for the EMG measurements. Each participant was also instructed on how to fit and wear the VR headset, and a short training session was offered to ensure participants were familiar with the self-rating task and understood what Arousal and Valence meant.

All participants watched the videos in the same order within each category. There was a 10 s break between each video, during which participants were asked to self-rate their emotional state after watching the video stimulus. Participants rated how they felt in terms of emotional valence and emotional arousal on a scale from one to nine. On the valence scale, one denoted a very unpleasant emotion, five denoted a neutral emotional state, and nine denoted a very pleasant emotion. On the arousal scale, one denoted very low arousal, five denoted a medium level of arousal, and nine denoted very high arousal.

### EMG data pre-processing

Surface EMG (sEMG) is electrophysiological recording technology used for the non-invasive detection of muscle excitation and activation^42^. The recorded signal represents the electrical activity generated by a contracting muscle, which can be detected by placing electrodes on the skin over the muscle of interest. In fact, the signal can be defined as the algebraic sum of the motor unit action potentials generated by the active motor units, which the electrodes detect over the skin^43^. In this study, the sEMG sensors were used to measure the electrical activity of motor units in the striated muscles of the face.

Spontaneous sEMG activity of the facial muscles generally has a small amplitude and may be contaminated by motion artifacts and nonmyogenic potentials associated with eye movements, endogenous eyeblinks, brain activity, retinal responses, and salivary gland activity^44^. Additionally, noise caused by electromagnetic interference can affect the sEMG signal. To increase the quality of the obtained sEMG signals, we performed signal de-noising. The idea is to improve the signal-to-noise ratio while causing as little distortion to the informative sEMG components as possible. The artifacts caused by motion and nonmyogenic potentials are generally limited to the low-frequency region of the EMG power spectrum (0–20 Hz region). These artifacts can be easily eliminated by using a high-pass filter. However, because the EMG power spectra of the facial muscles show relatively large real EMG components near the low-frequency region^45^, a cut-off point for each muscle should be carefully selected. In our work, we selected the cut-off points for the different muscles based on the work presented in^44^. We utilized a high-pass cut-off frequency of 30 Hz for the corrugator supercilii and a high-pass cut-off frequency of 20 Hz for the orbicularis oculi and the zygomaticus major. The noise caused by electromagnetic interference has visible components at 50 Hz and its harmonics. To reduce the influence of electromagnetic interference while retaining the informative sEMG components, we utilized a method based on spectrum interpolation^46^. The idea is that the magnitude of the frequency components affected by electromagnetic interference can be approximated by interpolating the signal’s amplitude spectrum using the magnitude of the stable neighboring components. Regarding the upper end of the sEMG frequency components, Boxtel et al.^44^ demonstrated that the signal power above 500 Hz is often negligible. Therefore, we employed a low-pass filter with a cut-off frequency of 500 Hz to isolate the uninformative part of the signal and ensure that unexpected artifacts in the signals would not appear.

Since voltage recorded from a muscle is difficult to describe in terms of level, interpreting muscle function directly from raw sEMG signal is challenging. As a result, researchers are primarily interested in the signal's amplitude. The amplitude of the sEMG signal has the potential to provide a measure of muscle force magnitude. In addition, the amplitude of a raw sEMG signal is often considered as the sum of the neural drive to the area where the electrode is positioned, which is related to the muscle’s excitement. Root mean square (RMS) and mean absolute value (MAV) are two commonly utilized methods for amplitude extraction. The RMS represents the square root of the average power of the EMG signal over a given period. The MAV represents the average rectified value, which is the area under the EMG signal once it has been rectified. It means that all the negative voltage values have been converted to positive voltage values. In this study, we analyzed the RMS, which is frequently chosen over the MAV value because it represents the power of the EMG signal.

The factors affecting the EMG signal differ across individuals, between days within an individual, and even within a day within an individual, in case the electrode setup has been altered. Given the large number of factors that influence the EMG signal, voltage recorded from a muscle is difficult to describe in terms of a level if there is no reference value to which it can be compared. As a result, interpreting the amplitude of the raw EMG signal is difficult unless normalization is performed. We performed person-specific min–max normalization. The minimum and the maximum values of the signals were calculated over a winsorized data distribution. Winsorization was applied to the EMG signals using their 5th and the 95th percentile. More specifically, all data points in the signal with a value lower than the value of the 5th percentile were set to 0, and all data points with a value higher than the value of the 95th percentile were set to the value of the 95th percentile. This was done to avoid insignificant EMG activations captured by the sensor and to avoid amplitude bursts. After the winsorization, the data were normalized between 0 and 1 per subject.

### Statistical analysis

The overall analysis was performed in Python in combination with the statannot library (version 0.4.4, https://pypi.org/project/statannot/). We did hypothesis testing using the Wilcoxon signed-rank test—a test with a null hypothesis that two related paired samples come from the same distribution. It tests whether the distribution of the differences x − y is symmetric around zero. It is a non-parametric version of the paired T-test. The *p*-values were Bonferroni-corrected. The value of *n* was set to the number of comparisons performed in the specific experiment (*n* = 6 for the subjective valence and arousal experiments presented in Figs. 4 and 6; *n* = 18 for the experiments on predefined video type presented in Fig. 7). The significance annotations were represented as: * if *p* $\in$ [0.05, 10^−2^); ** if *p* $\in$ [10^−2^, 10^−3^); *** if *p* $\in$ [10^−3^, 10^−4^); and **** *if p* ≥ 10^−4^.

## Supplementary Information

Supplementary Information.

## Data Availability

The data and the code are publicly available at: https://github.com/emteqlabs/EmgDataVR. The data includes raw 1000 Hz EMG signals and pre-processed EMG signals, ready for statistical analysis.
