# Supplementary material for: Facial EMG sensing for monitoring affect using a wearable device
Source: Sci Rep. 2022 Oct 7;12:16876. doi: 10.1038/s41598-022-21456-1 (PMC9542454; doi:10.1038/s41598-022-21456-1)
Supplement: Supplementary file 1 — Supplementary Information. [file 41598_2022_21456_MOESM1_ESM.pdf]

Facial EMG sensing for monitoring affect using a wearable device

Supplementary Information

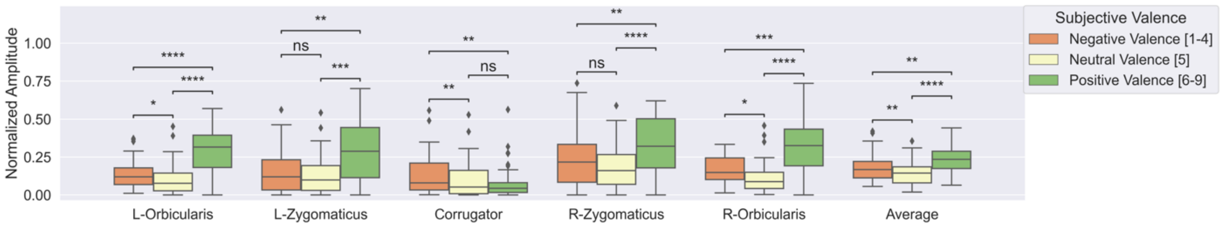

*Figure S. 1. Wilcoxon signed-rank (paired) test with Bonferroni correction for videos with negative valence ratings (1-4) vs. videos with neutral valence ratings (rating 5) vs. videos with positive valence ratings (6-9), applied for each of the five sensors (left orbicularis, left zygomaticus, corrugator, right zygomaticus, and right orbicularis) and the average amplitude of all five sensors. Statistical significance annotations: * if p ∈ [.05, 10-2); ** if p ∈ [10-2, 10-3); *** if p ∈ [10-3, 10-4); and **** if p >= 10-4.*

Table S. 1. Demographic information for each participant in the study.

Participant ID	Age	Gender	Participant ID	Age	Gender
1	30	female	21	47	male
2	31	male	22	19	female
3	29	male	23	18	male
4	23	female	24	53	female
5	23	female	25	27	male
6	24	male	26	26	male
7	29	male	28	31	female
8	33	male	29	47	male
9	23	male	30	59	female
11	27	male	31	57	male
12	24	male	32	22	male
13	18	female	33	29	male
14	27	female	34	26	male
15	35	male	35	26	male
16	68	female	36	54	female
17	64	male	37	31	female
18	31	male	38	34	male
19	27	female	39	26	male
20	55	male	41	19	female

Table A. 2 List of selected video stimuli used in our study and corresponding validation studies.

Video ID	Category	Name	Validation participants	Validation study
1	Neutral	neu_airport2	411, 82, 38	[1] [2], this study
2	Neutral	neu_cafe	411, 82, 38	[1] [2], this study
3	Neutral	neu_assembly	411, 82, 38	[1] [2], this study
4	Neutral	neu_sanfran	411, 82, 38	[1] [2], this study
5	Neutral	neu_cityinthenight	411, 82, 38	[1] [2], this study
6	Negative	neg_crocbitesman	411, 82, 38	[1] [2], this study
7	Negative	neg_bikefalloffcliff	411, 82, 38	[1] [2], this study
8	Negative	neg_bullthrownandtrample	411, 82, 38	[1] [2], this study
9	Negative	neg_carhitsskater	411, 82, 38	[1] [2], this study
10	Negative	neg_breakdancerkickskid	411, 82, 38	[1] [2], this study
11	Negative	neg_fish	86, 38	[3], this study
12	Neutral	neu_pillow	411, 82, 38	[1] [2], this study
13	Neutral	neu_ridingthetube2	411, 82, 38	[1] [2], this study
14	Neutral	neu_boydrinkingtea	411, 82, 38	[1] [2], this study
15	Neutral	neu_ridinginthetube1	411, 82, 38	[1] [2], this study
16	Neutral	neu_hikinginwood	411, 82, 38	[1] [2], this study
17	Positive	pos_cat_icecream	38	This study
18	Positive	pos_dog_nails	38	This study
19	Positive	pos_pixar	38	This study
20	Positive	cat_outfit	38	This study
21	Positive	pos_catlamp	38	This study
22	Positive	pos_cat_door	38	This study
23	Positive	pos_dogdownstairs	38	This study
24	Positive	pos_bridelauphingduringvows	411, 82, 38	[1] [2], this study
25	Positive	pos_babydancebeyonce	411, 82, 38	[1] [2], this study

References

1. Samson, AC, Kreibig, SD, Soderstrom, B, Wade, AA, & Gross, JJ, (2016), Eliciting positive, negative and mixed emotional states: A film library for affective scientists. Cognition and Emotion, **30(5)**, pp. 827-856, (2016).
2. Mavridou I, Balaguer-Ballester E, Seiss E, & Nduka C. Affective State Recognition in Virtual Reality from Electromyography and Photoplethysmography using Head-mounted Wearable Sensors. Doctorate Thesis, Bournemouth University. (2021).
3. Gnacek M., Mavridou I, Seiss E, Kostoulas T, Balaguer-Ballester E., Nduka C. "AVDOS -Affective Video Database Online Study." 2022, In: 10th International Conference on Affective Computing and Intelligent Interaction (ACII), Japan, 18-21 October, p:8, (2022).
